# Optimization of *trans*-4-hydroxyproline synthesis pathway by rearrangement center carbon metabolism in *Escherichia coli*

**DOI:** 10.1186/s12934-023-02236-6

**Published:** 2023-11-20

**Authors:** Yu Gong, Ruiqi Wang, Ling Ma, Shuo Wang, Changgeng Li, Qingyang Xu

**Affiliations:** 1 College of Biotechnology, Tianjin University of Science & Technology, Tianjin, 300457 People’s Republic of China; 2 Key Laboratory of Industrial Fermentation Microbiology, Ministry of Education, Tianjin University of Science & Technology, Tianjin, 300457 People’s Republic of China

**Keywords:** *Escherichia coli*, *trans*-4-Hydroxyproline, Acetyl-CoA, NADPH supply, NOG pathway, α-Ketoglutarate

## Abstract

**Background:**

*trans*-4-Hydroxyproline (T-4-HYP) is a promising intermediate in the synthesis of antibiotic drugs. However, its industrial production remains challenging due to the low production efficiency of T-4-HYP. This study focused on designing the key nodes of anabolic pathway to enhance carbon flux and minimize carbon loss, thereby maximizing the production potential of microbial cell factories.

**Results:**

First, a basic strain, HYP-1, was developed by releasing feedback inhibitors and expressing heterologous genes for the production of *trans*-4-hydroxyproline. Subsequently, the biosynthetic pathway was strengthened while branching pathways were disrupted, resulting in increased metabolic flow of α-ketoglutarate in the Tricarboxylic acid cycle. The introduction of the NOG (non-oxidative glycolysis) pathway rearranged the central carbon metabolism, redirecting glucose towards acetyl-CoA. Furthermore, the supply of NADPH was enhanced to improve the acid production capacity of the strain. Finally, the fermentation process of T-4-HYP was optimized using a continuous feeding method. The rate of sugar supplementation controlled the dissolved oxygen concentrations during fermentation, and Fe^2+^ was continuously fed to supplement the reduced iron for hydroxylation. These modifications ensured an effective supply of proline hydroxylase cofactors (O_2_ and Fe^2+^), enabling efficient production of T-4-HYP in the microbial cell factory system. The strain HYP-10 produced 89.4 g/L of T-4-HYP in a 5 L fermenter, with a total yield of 0.34 g/g, the highest values reported by microbial fermentation, the yield increased by 63.1% compared with the highest existing reported yield.

**Conclusion:**

This study presents a strategy for establishing a microbial cell factory capable of producing T-4-HYP at high levels, making it suitable for large-scale industrial production. Additionally, this study provides valuable insights into regulating synthesis of other compounds with α-ketoglutaric acid as precursor.

**Supplementary Information:**

The online version contains supplementary material available at 10.1186/s12934-023-02236-6.

## Background

Trans-4-hydroxyproline (T-4-HYP) is an imino acid [1] and the primary constituent of collagen [2]. It has extensive applications in various fields, such as biomedicine, chemical manufacturing, food nutrition, and the beauty, and skincare sectors [3]. T-4-HYP is a crucial raw material for the synthesis of carbapenem antibiotics, angiotensin-converting enzyme inhibitors, and other pharmaceuticals [4]. Additionally, it possesses antitumor, immunosuppressive, anti-HIV, and anti-inflammatory properties. Its versatility has led to a global increase in its use. The conventional method for synthesizing T-4-HYP involves a complex and expensive process of acid-base or protease hydrolysis of animal proteins [5]. With the development of biotechnology, microbial fermentation has gradually replaced traditional production processes owing to its advantages of high efficiency, environmental sustainability, and cost-effectiveness. However, compared to other products, the fermentation control of T-4-HYP is complicated, it remains challenging to develop microbial strains that meet the requirements of industrialization and commercialization.

Recently, numerous researchers have attempted to develop a rapid and efficient transhydroxyproline synthesis pathway for microbial production, with promising results. Shibasaki et al. [6] screened and identified a highly active proline-4-hydroxylase (P4H) from *Cystomyces*. Using proline as a substrate, they achieved a T-4-HYP concentration of 41.0 g/L within 100 h, demonstrating the potential for the microbial production of T-4-HYP. In another study, Shibasaki et al. introduced the proline-4-hydroxylase gene into L-Pro-producing *Escherichia coli* and produced 25.0 g/L T-4-HYP at 96 h [7]. Lawrence et al. [8] reported thatα-ketoglutarate and Fe^2+^ were necessary for proline hydroxylation in vitro. As key intermediate of the tricarboxylic acid cycle (TCA cycle) in *E. coli*, α-ketoglutarate can directly use glucose and proline to produce T-4-HYP, without requiring any extra addition [9]. Therefore, in the process of proline hydroxylation, the regulation of the TCA cycle, which is the main production pathway of α-ketoglutarate, is also very important [7]. Falcioni et al. [10] introduced strain RH1 into the proline-producing isoleucine-growth retardation strain C and produced 7.1 g/L T-4-HYP at a molar ratio of 46:1 (glucose/isoleucine). Within 23 h, the proline yield reached 98.5%. Wang et al. [11] constructed a high-yield HYP *E. coli* NA45 strain, following sequence optimization, vector screening, and NaNO2-ARTP combination mutagenesis, using glycerol as the carbon source, and produced 25.4 g/L T-4-HYP in a fed-batch culture within 48 h. In the same year, Zhang et al. [2] knocked out key genes (*PutA*, *SucAB*, *AceA*, and *AceK* ) involved in the metabolic processes to couple cell growth with the Hyp synthesis pathway and produced 31.0 g/L trans-Hyp using glucose as a substrate through a batch feeding strategy. Long et al. [12] synthesized a T-4-HYP high-producing strain de novo using codon selection evolution, quorum-sensing tunable circuit lamps, and other strategies, and accumulated 54.8 g/L T-4-HYP within 60 h of growth, this is the highest yield reported so far. However, the problem of low yield due to carbon loss in the process of microbial production of T-4-HYP hinders the large-scale industrial production of T-4-HYP. Acetyl-CoA is formed by the oxidative decarboxylation of pyruvate, and the inherent carbon loss associated with this process significantly limits the yield of acetyl-CoA derivatives in commonly used industrial platform strains. Wei et al. [13] used both glucose and xylose as carbon sources to produce 4-Hydroxyisoleucine (4-HIL), and introduced the Weimberg pathway to minimize the carbon loss, resulting in a decrease in sugar consumption of 24.5%, an increase in yield by 31.6%. A final 4-HIL yield of 29.16 g/L was achieved. Therefore, it is an important step in microbial production to maximize carbon flux by coordinating all aspects of the metabolic pathway.

**Fig. 1:**
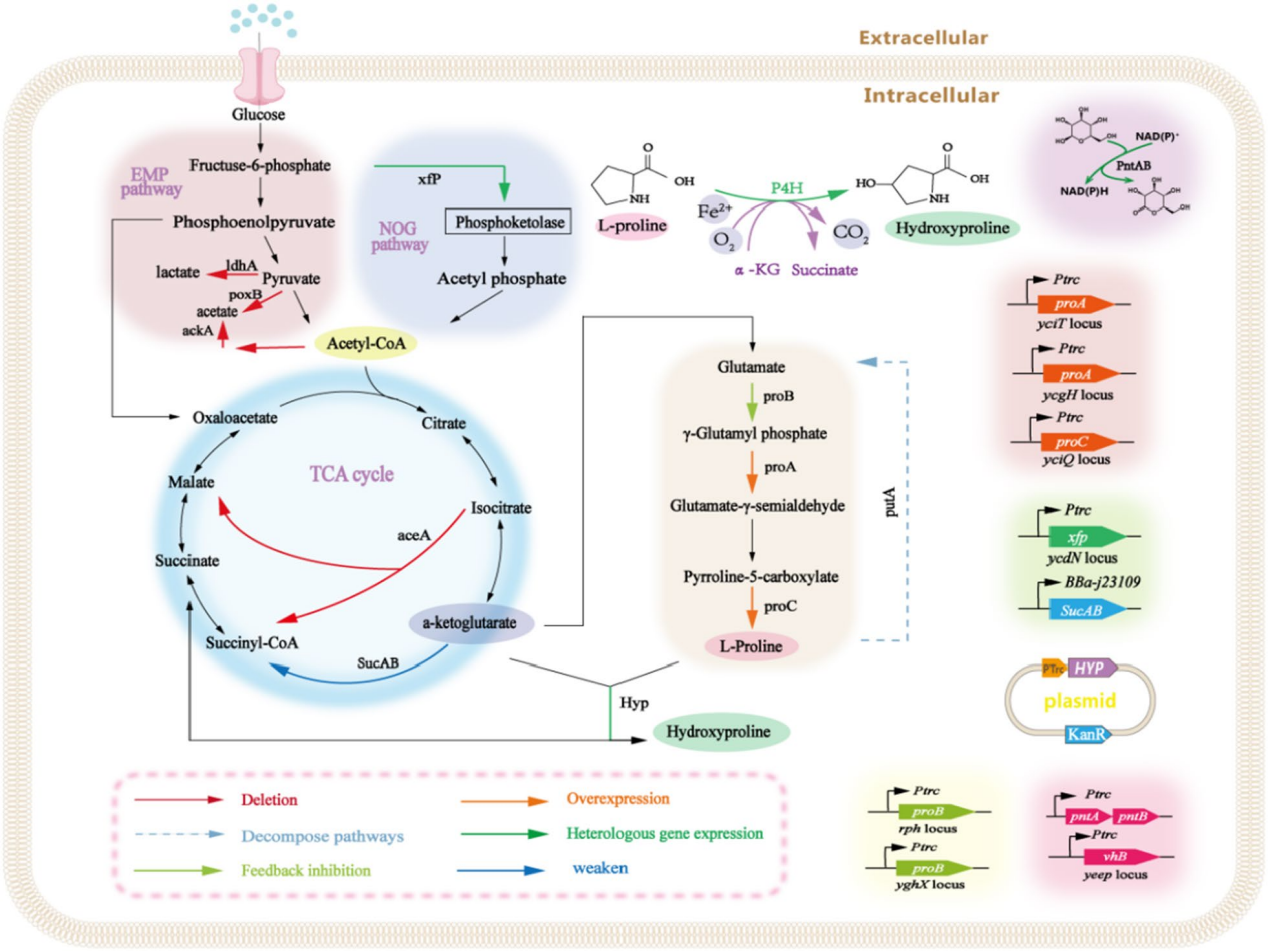
*trans*-4-Hydroxyproline metabolic pathway of central carbon metabolic rearrangement in *Escherichia coli.* The orange arrow indicates that the endogenous gene is overexpressed. Red arrow indicates that the related genes have been deleted. Green arrow indicates the introduction of heterologous genes. Yellow green indicates the gene that relieves feedback inhibition. The solid blue line indicates a weakened gene. The blue dotted line represents genes involved in the decomposition pathway. Hyp: from *Dactylosporangium* sp. and Xfp: from *Bifidobacterium adolescentis*

Owing to the complex distribution of metabolic flux in the T-4-HYP synthesis pathway and the strict requirements of fermentation conditions, large-scale industrial production has not been realized. The primary issues are as follows: In *E. coli*, increased metabolic shunts lead to considerable carbon loss and an insufficient supply of reducing power, and cofactors (O_2_, Fe^2+^) are difficult to supplement effectively during fermentation. Therefore, it is particularly important to rationally design carbon metabolism and key nodes of the T-4-HYP synthesis pathway, redistribute anabolic flow, and establish an efficient microbial cell factory to produce high yields of T-4-HYP. The metabolic pathway of T-4-HYP and the process of strain construction are shown in Fig. 1. First, *E. coli W3110* was used to construct a basic strain for the synthesis of T-4-HYP, and the heterologous expression of proline hydroxylase in *E. coli* cells was observed. The metabolic flux of α-ketoglutarate in the TCA cycle was enhanced by key point modifications, and the heterologous phosphoenolketolase (NOG) pathway was introduced to redirect the carbon flux from glucose to acetyl-CoA, which enhanced the carbon flux of the TCA cycle. An NADPH-enhanced module was constructed to provide sufficient reducing power for the intermediate metabolic processes. Finally, the dissolved oxygen levels during fermentation was controlled by adjusting the glucose flow acceleration rate to enhance the intracellular oxygen concentration. Reduced iron was supplemented by continuously feeding an Fe^2+^ solution for the hydroxylation process. According to the efficient fermentation strategy established in this study, the strain could produce 89.4 g/L of T-4-HYP in a 5 L bioreactor with a yield of 0.34 g/g glucose. This microbial fermentation method described in this study achieves the highest yield of T-4-HYP and is both an economical and efficient fermentation process. It has been demonstrated that the introduction of the NOG pathway can greatly increase the synthesis of T-4-HYP. This metabolic engineering strategy can be applied for the effective production of compounds using α-ketoglutarate as a precursor. These results lay the foundation for large-scale production of T-4-HYP.

## Results

### Construction of l-proline chassis strain

**Fig. 2:**
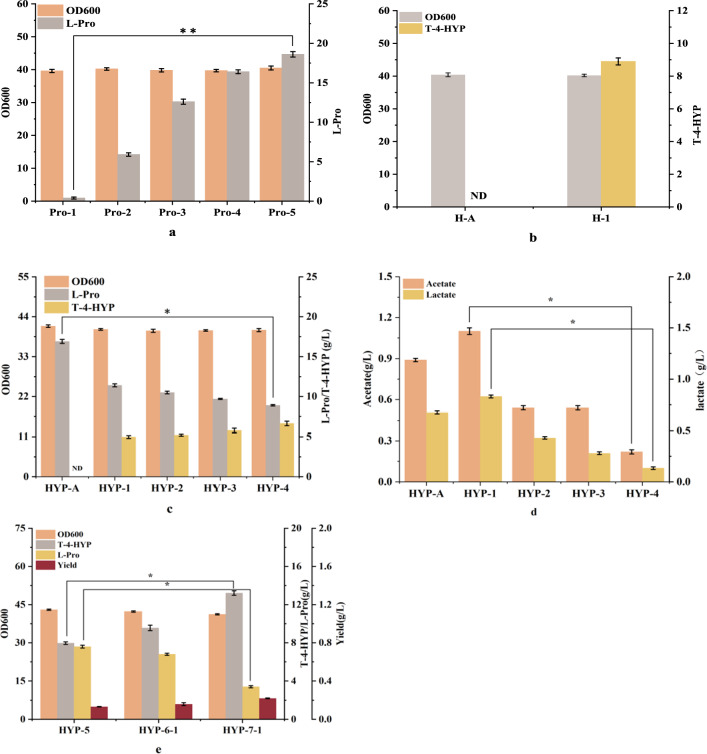
**a** Shake flask fermentation results of strain Pro-1–Pro-5. **b** Effects of T-4-HYP expression elements on T-4-HYP fermentation. **c** Effects of enrichment precursors on T-4-HYP production and cell growth. **d** The effect of enrichment precursors on by-products. **e** Shake flask fermentation studies of strain HYP-5–HYP-7-1

To enable the engineered strain of *E. coli* to de novo synthesize T-4-HYP from glucose, significant accumulation of the precursor l-proline (L-Pro) is necessary. In *E. coli*, proline is catabolized to glutamate by proline dehydrogenase (*PutA*) [14]. Previous studies [9] have shown that the deletion of *putA* increases the accumulation of L-Pro. Therefore, we deleted the *putA* gene in the *E. coli* W3110 genome, inhibiting the pathway for L-Pro degradation. The resulting strain, Pro-1, accumulated 1.39 g/L of L-Pro after 26 h of shake flask fermentation (Fig. 2a).

Next, we deleted lacI from the Pro-1 strain to ensure constitutive expression of genes controlled by the Ptrc promoter. Feedback inhibition and overexpression of key metabolic nodes in the pathway are important and effective strategies for overproducing L-Pro [15, 16]. L-Pro synthesis from glutamate involves three enzymatic reactions (Fig. 1). The first step involves the conversion of glutamate to γ-glutamyl phosphate by γ-glutamyl kinase, and allosteric inhibition of γ-glutamyl kinase activity leads to the breakdown of proline into glutamate. This reaction is catalyzed by the γ-glutamyl kinase encoded by the *proB* gene [15], which is the rate-limiting enzyme in the synthesis [15]. It has been reported [15] that the mutant gene *proB*_*74*_ effectively relieved feedback inhibition by replacing the 319th G in proB with an A, resulting in the substitution of asparagine for aspartic acid at position 107 of the predicted protein. Therefore, the *proB*_*74*_ gene controlled by a strong Ptrc promoter was integrated into the pseudogene *yghX* locus of Pro-1, forming the Pro-2 (Table 1), which produces 5.9 g/L of L-Pro. This increased the yield 4.2 times higher than that of the control strain Pro-1 (1.39 g/L), confirming that the mutant gene *proB*_*74*_ alleviated feedback inhibition caused by proline accumulation, which is consistent with previous research [15]. In order to enhance the metabolic flow of proline,the *ProB*_*74*_ was integrated again into the pseudogene rpH locus of Pro-2 to generate Pro-3 while being controlled by the Ptrc promoter (Table 1). The shake flask data for *E. coli* are shown in Fig. 2a, with Pro-3 accumulating 10.6 g/L of L-Pro. Glutamate-γ-semialdehyde dehydrogenase (*proA*) catalyzes the second step reaction, which catalyzes the conversion of γ-glutamyl phosphate to glutamate-γ-semialdehyde. In order to enhance L-Pro metabolic flux, the gene *proA* controlled by a strong promoter Ptrc was integrated into the pseudogene *yjiT* locus of Pro-3 to obtain Pro-4 (Table 1). As shown in Fig. 2a, after 26 h of fermentation, Pro-4 produced 13.4 g/L L-Pro, L-Pro metabolic flux was significantly enhanced. Pyrroline-5-carboxylate reductase (*proC*) catalyzes the third step reaction, so the *ProC* gene controlled by a strong Ptrc promoter was integrated into the pseudogene *yciQ* locus of Pro-4 (Table 1), forming the pro-5. As shown in Fig. 2a, the yield of L-Pro was 15.6 g/L, and the metabolic flux of L-Pro was strengthened again, which proved that overexpression of *proC* gene promoted the biosynthesis of L-Pro. At the same time, there was no significant difference in the biomass of strains Pro-1 to Pro-5 (Fig. 2a), indicating that cell growth was not affected. This result confirmed that the metabolic flux of the L-Pro biosynthetic pathway in *E. coli* was effectively enhanced by removing feedback inhibition and strengthening key genes.

### Construction of *trans*-4-hydroxyproline expression element and its introduction into the l-proline chassis strain

The key determinant for converting l-proline (L-Pro) into T-4-HYP is the activity of proline hydroxylase, which plays a pivotal role in enhancing hydroxylation efficiency and subsequently increasing T-4-HYP production rates. Therefore, we chose a previously reported *trans*-Proline-4-hydroxylase gene (DaP4H) from *Dactylosporangium* sp. [6]. To ensure its successful expression in *E. coli*, we subjected DaP4H to codon optimization and subsequently integrated it into plasmid Ptrc99a, resulting in the Ptrc-DaP4H construct. Upon successful transformation into the wild-type *E. coli* strain W3110, the engineered strain H-1 was generated. In parallel, an empty Ptrc99a plasmid was introduced into the wild-type *E. coli* W3110 strain to generate the control strain, H-A. Both strains were subjected to shake-flask fermentation using a substrate mixture of 10.0 g/L proline and 2.0 g/L α-ketoglutarate for 26 h. Notably, the H-1 strain exhibited a considerable T-4-HYP yield of 8.9 g/L, whereas the control strain H-A did not produce any T-4-HYP (Fig. 2b). However, the fact that both strains exhibited nearly identical optical densities at 600 nm (OD600), confirmed the efficient expression of the heterologous proline hydroxylation element Ptrc-DaP4H in *E. coli* W3110, with no discernible impact on cellular growth.

Furthermore, we introduced the Ptrc-DaP4H plasmid into the Pro-5 strain, resulting in the recombinant strain HYP-1. Concurrently, empty Ptrc99a plasmid was introduced into the Pro-5 strain to generate the control strain, HYP-A. The results of 26 h shake flask fermentation are presented in Fig. 2c. Remarkably, strain HYP-1 produced a substantial amount of T-4-HYP (4.98 g/L), whereas strain HYP-A did not produce T-4-HYP but accumulated 16.9 g/L of L-Pro. Importantly, both strains displayed comparable OD values at 26 h, confirming the successful transformation of endogenously produced L-Pro into T-4-HYP and the non-interference of the introduced T-4-HYP expression element with cellular growth. Intriguingly, strain HYP-1 also produced 11.4 g/L of L-Pro, which was not effectively converted into T-4-HYP.

Additionally, 1.1 g/L of acetate and 0.83 g/L of lactate were produced (Fig. 2d), signifying the concurrent diversion of carbon metabolic flux towards acetate biosynthesis during the T-4-HYP synthesis pathway.

The genotype of strains: Pro-1, *E. coli W3110*, ∆putA; Pro-2, Pro-1 ∆*lacI*, *yghX*::P_*trc*_-*proB*_*74*_; Pro-3, Pro-2 *rpH*::P_*trc*_-*proB*_*74*_; Pro-4, Pro-3 *yjiT*::P_*trc*_-*proA*; Pro-5, Pro-4 *yciQ*::P_*trc*_-*proC*; H-1, *E. coli* W3110 P_*trc*_-DaP4H; H-A, *E. coli* W3110 Ptrc99a; HYP-A, Pro-5 Ptrc99a; HYP-1, Pro-5, Ptrc99a-DaP4H; HYP-2, HYP-1, ∆*ldhA*; HYP-3, HYP-2, *∆poxB*; HYP-4, HYP-3 *∆ackA*; HYP-5, HYP-4, *∆aceA*.

Data are expressed as mean and error bars are expressed as standard deviation (n = 3 independent experiments). *Means 0.01 < P < 0.05, **means P < 0.01.

### Enhancing the metabolic flux of the precursor to increase the production of *trans*-4-hydroxyproline

In *E. coli*, acetyl-CoA and α-ketoglutarate serve as crucial central intermediates in the T-4-HYP biosynthetic pathway [17, 18]; however, they are also key bottlenecks in efficient T-4-HYP production. To address this, we strategically inhibited the side pathways of pyruvate to eliminate byproduct formation and enhance pyruvate availability. Specifically, we disrupted the lactate dehydrogenase gene (*ldhA*) to impede lactate synthesis, resulting in strain HYP-2 (Fig. 2c), which exhibits a modest 6.2% increase in pyruvate accumulation (5.29 g/L) compared to the control strain HYP-1. Additionally, PoxB can convert pyruvate into acetic acid and CO_2_, deleted (*PoxB*) to generate the strain HYP-3 in order to prevent acetate production. HYP-3 yielded 0.35 g/L of acetate and achieved a T-4-HYP titer of 5.76 g/L, representing a 9.0% improvement over HYP-2. However, pyruvate-to-acetate conversion catalyzed by acetyl-CoA via acetate kinase A (*AckA*) leads to carbon loss, which is detrimental to fermentation [19]. Thus, *ackA* was knocked out to obtain the strain HYP-4. As shown in Fig. 2c, HYP-4 accumulated merely 0.22 g/L of acetate, 59% less than HYP-3. The augmented acetyl-CoA pool in HYP-4 contributed to an enhanced T-4-HYP production of 6.62 g/L, 14.9% increase compared to HYP-3 (Fig. 2c). These results unequivocally demonstrate that *ackA* knockout significantly reduces acetate byproduct formation. Isocitrate lyase is encoded by the *aceA* gene, which can directly convert isocitrate into succinic and malic acids through the glyoxylic acid cycle and is a competitive pathway for the synthesis of α-ketoglutarate. Therefore, *aceA* was deleted to prevent the loss of isocitrate and improve the metabolic flow to α-ketoglutarate. Deletion of the *aceA* gene produced the strain HYP-5, and the yield of T-4-HYP reached 7.97 g/L (Fig. 2e), which was 17.6% higher than that of strain HYP-4,The yield increased significantly, and the yield of HYP-7-1 was 0.22 g/g, which is 66.7% higher than that of HYP-5.

**Fig. 3:**
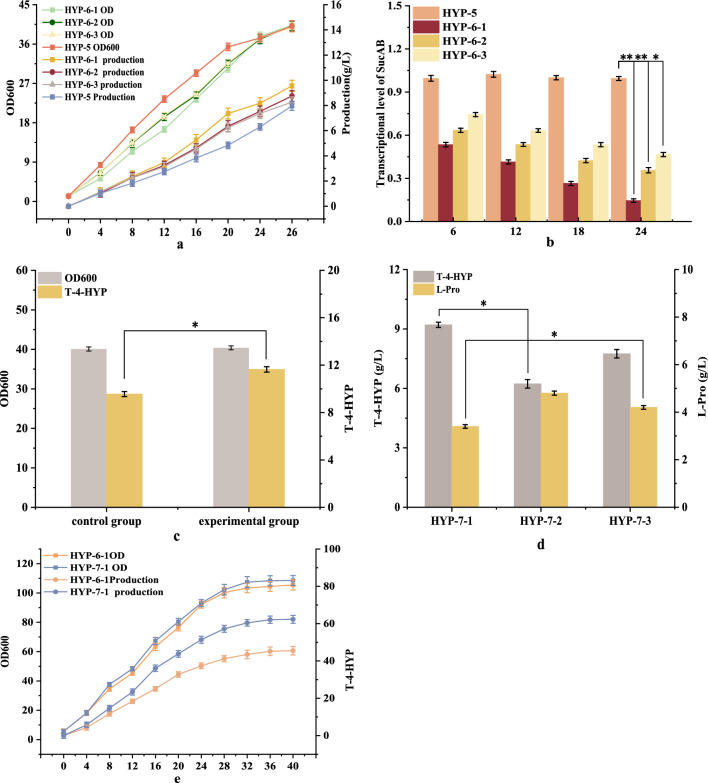
**a** Effect of *SucAB* gene controlled by different promoters on T-4-HYP fermentation. **b** Relative transcription level of *SucAB* gene in four strains with different culture times. **c** Effect of adding α-ketoglutrate on T-4-HYP fermentation. **d** Effect of *xfp* gene from different sources on T-4-HYP production. **e** Comparison of fermentation performance between strains HYP-6-1 and HYP-7-1

α-Ketoglutarate plays a pivotal role in the T-4-HYP biosynthetic pathway, serving as an intermediate in the TCA cycle and as a precursor for T-4-HYP biosynthesis (Fig. 1). Chen et al. [20] found that the *E. coli* host does not have enough α-ketoglutarate to supply the *E. coli* host, resulting in Hyp cannot be efficiently produced. Considering the pivotal role of central carbon metabolism in both cell growth and T-4-HYP biosynthesis, the precise carbon flux at the α-ketoglutarate node must be tailored to achieve a harmonious equilibrium between these two processes. To achieve this, we introduced weak promoters of varying strengths upstream of *sucAB* into strain HYP-5 to modulate *sucAB* transcription. The weak promoters BBa-j23109, BBa-j23115, and BBa-j23114 resulted in strains HYP-6-1, HYP-6-2, and HYP-6-3, respectively. After 26 h of shake flask fermentation (Fig. 3a), all three engineered strains HYP-6-1, HYP-6-2, and HYP-6-3 displayed elevated T-4-HYP titers, reaching 9.56 g/L, 8.75 g/L, and 8.27 g/L, respectively, compared to the control strain HYP-5. Although these strains exhibited slower growth than HYP-5 during the initial stages, their later growth converged to similar levels with no significant differences in biomass. As L-Pro was converted to T-4-HYP, α-ketoglutarate was directed towards succinate through the glyoxylate bypass, thus facilitating cellular growth restoration, consistent with prior research findings [2]. Analysis of the relative transcription levels of *SucAB* at different fermentation time points (Fig. 3b) corroborated the successful downregulation of *SucAB* gene expression using different weak promoters. BBa-j23109 exhibited the most pronounced effect. By comparing Fig. 3a, it was evident that fine-tuning the expression of *sucAB* effectively improved T-4-HYP biosynthesis without significantly compromising *E. coli* growth.

These genetic modifications effectively alleviate the metabolic flux bottleneck in T-4-HYP biosynthesis. Nonetheless, a residual amount of 6.5 g of L-Pro remained (Fig. 2e). To address this, we explored the addition of 2.0 g/L of α-ketoglutarate to the existing culture medium to investigate whether exogenous α-ketoglutarate supplementation could enhance T-4-HYP synthesis. After 26 h of shake flask fermentation, the results (Fig. 3c) demonstrated that the addition of α-ketoglutarate led to a remarkable increase in T-4-HYP production, reaching 11.64 g/L, signifying a 21.7% increase over the control group without adding α-ketoglutarate. This outcome underscores the need for further reinforcement of α-ketoglutarate metabolism, and the subsequent introduction of the NOG pathway is anticipated to further intensify carbon flux towards α-ketoglutarate.

The genotype of strains: HYP-6-1, HYP-5 P_*BBa-j23109*_-*SucAB*; HYP-6-2, HYP-5 P_*BBa-j231115*_-*SucAB*; HYP-6-3, HYP-5 P_*BBa-j23114*_-*SucAB*; HYP-7-1, HYP-6-1 ycdN::P_*trc*_-*Baxfp*; HYP-7-2, HYP-6-1 ycdN::P_*trc*_-*Acxfp*; HYP-7-3, HYP-6-1 ycdN::P_*trc*_-*Blxfp*.

Data are expressed as mean and error bars are expressed as standard deviation (n = 3 independent experiments). *Means 0.01 < P < 0.05, **means P < 0.01.

### Introducing the NOG pathway to reduce carbon loss and increase yield

**Fig. 4:**
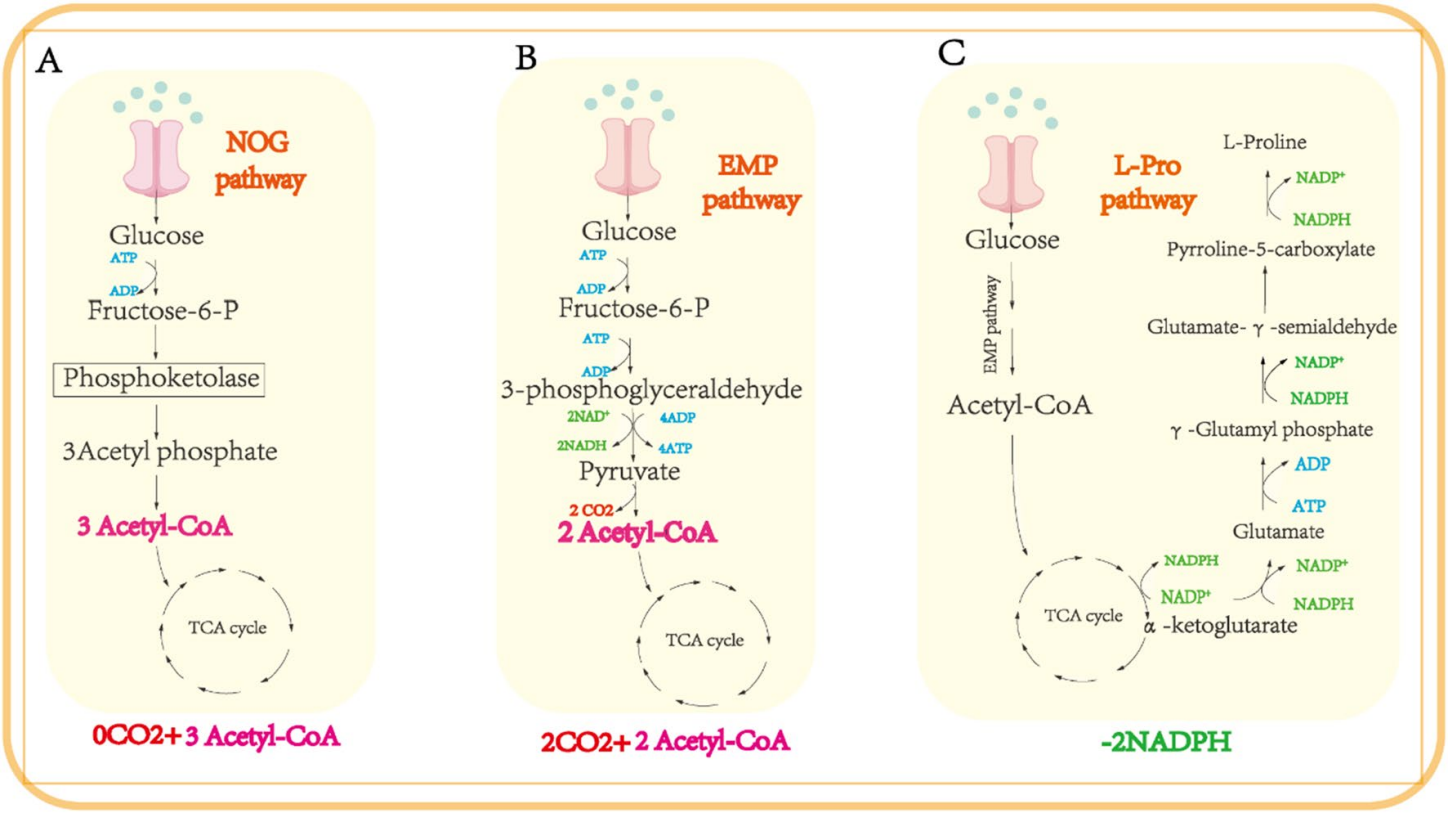
The structures of NOG, EMPand L-pro synthesis pathways; **a **the simplified scheme of non-oxidative glycolysis; **b** a simplified EMP (glycolysis) scheme; **c** L-pro synthesis pathway a: Glucose → 3 acetyl-CoA; b: Glucose → 2 acetyl-CoA + 2CO_2_. *EMP pathway* Embden–Meyerhof–Parnas, *NOG pathway* non-oxidative glycolysis, *fructose-6-P* fructose 6-phosphate

The glycolytic pathway is the basic pathway of glucose catabolism in cells and converts external glucose into a carbon source for use in the body. Pyruvate decarboxylase catalyzes the conversion of pyruvate to acetyl-CoA via a series of catalytic reactions using glucose as the starting material. This process loses CO_2_, resulting in carbon loss and limiting the yield of the target product [21] (Fig. 4b). However, phosphoketolase (encoded by *xfp*), the key enzyme in the NOG pathway, is mainly present in the phosphoketolase pathway of heterolactic fermentation and shunt metabolism in *Bifidobacterium* [22]. As shown in Fig. 4a, *xfp* weakens the Embden–Meyerhof–Parnas (EMP) pathway during expression, converting one molecule of glucose into three molecules of acetyl-CoA without CO_2_ emissions. The theoretical carbon yield of acetyl-CoA increased from 0.66 mol mol mol^−1^ to 1.00 mol mol mol^−1^ [23], and carbon is totally preserved in glycolytic metabolism, similar to acetyl-CoA. Enhanced carbon flux in the TCA cycle. Heterologous introduction of this pathway into other engineered bacteria can significantly increase product yield using acetyl-CoA as a precursor [22]. Kanokarn et al. [24] used strains wketoglutaric acid, the yield of hydroxyproline was greatly improved [25]. The phosphoketolase pathway exists in fungi and several bacteria and shows different substrate specificities and expression intensities among different species [26]. Therefore, we selected three reported xfps for comparative experiments. These were isolated from *Bifidobacterium adolescentis* (Gene ID:56674845), *Clostridium acetobutylicum* (GenBank: KHD36088.1), and *Bifidobacterium longum* (NCBI-Gene ID:56674845). The three genes, *Baxfp*, *Acxfp*, and *Blxfp*, which are controlled by the Ptrc promoter, were codon optimized and integrated into the *ycdN* locus of the HYP-6-1genome, strains HYP-7-1, HYP-7-2, and HYP-7-3 were obtained, respectively. Strain HYP-6-1 was used as the control for shake-flask fermentation. Data from the *E. coli* shake flask fermentation are shown in Fig. 3d. After 26 h of fermentation, the T-4-HYP yields in the HYP-7-1, HYP-7-2, and HYP-7-3 groups were 13.21, 10.52, and 11.42 g/L, respectively. The yield from strain HYP-7-1 was 30% higher than that of HYP-6. This demonstrates that engineered *E. coli* cells can convert glucose through the NOG pathway with sufficient biotransformation activity and that *Baxfp* is more suitable for the expression of T-4-HYP in *E. coli*. The production performance of strains HYP-6-1 and HYP-7-1 was tested in a 5 L fermenter. As shown in Fig. 3e, the yields of T-4-HYP were 45.5 and 62.4 g/L, respectively, and in strain HYP-7-1, the yield of T-4-HYP from glucose was 0.28 g/g. Introduction of the NOG pathway increased carbon flux to the TCA cycle, and the yield of T-4-HYP was greatly improved. This finding is consistent with those of the previous studies. Strain HYP-7-1 uses glucose as a carbon source to supply acetyl-CoA for high T-4-HYP yields.

### Enhancing NADPH supply

**Fig. 5:**
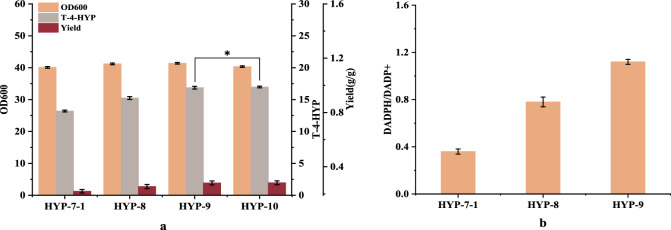
Shake flask fermentation parameters of strains HYP-7-1, HYP-8, HYP-9, and HYP-10 (**a**), and intracellular NAD(P)H/NAD(P) ratio of strains HYP-7-1, HYP-8, and HYP-9 (**b**). HYP-8, HYP-7-1, P_*trc*_-*PntAB*; HYP-9, HYP-8,*yjiP*::P_*trc*_-*PntAB*; data are expressed as mean and error bars are expressed as standard deviation (n = 3 independent experiments). *Means 0.01 < P < 0.05, **means P < 0.01

NADPH provides a large amount of reducing power for the growth and metabolism of various organisms. Fuhrer et al. [27] reported that the in vivo demand for NADPH in biosynthesis far exceeds the amount of NADPH produced. To maintain growth, the catabolism of NADPH should be balanced with the demand for the anabolism of the target products. In this study, 3 mol of NADPH was required for the synthesis of 1 mol of L-Pro by acetyl-CoA (Fig. 4c), but only 1 mol of NADP^+^ was reduced to NADPH during this process. Therefore, an increase in the NADPH/NADP^+^ ratio in cells promotes the synthesis of T-4-HYP. Thus, additional methods are required to synthesize NADPH for cellular use. The *E. coli* membrane-bound pyridyl nucleotide transhydrogenase encodes *PntAB*, which is oxidized by NADH to NAD^+^ to drive the reduction of NADP^+^ to NADPH [28]. The strategy of maintaining intracellular NAD(P)H balance by enhancing the expression of *PntAB* to promote the synthesis of target products has proven to be simple and effective [29, 30]. In this study, the natural promoter of *pntAB* was replaced with the Ptrc promoter to improve its expression and obtain enhanced HYP-8 yields. The T-4-HYP yield of strain HYP-8 reached 15.24 g/L, which was approximately 15.3% (Fig. 5a) higher than that of the control HYP-7-1 strain. In addition, we introduced a second *pntAB* gene controlled by the Ptrc promoter to the yeeL site of HYP-8 and obtained the strain HYP-9, which produced 16.85 g/L of T-4-HYP (Fig. 5a) and was 10.5% higher than that of strain HYP-8. Intracellular reduced cofactor levels in the two strains were detected, and the total amount of NAD(P)H in HYP-9 was significantly higher than that in HYP-8 (Fig. 5b). These results indicate that *pntAB* overexpression has a positive effect on T-4-HYP production and cell growth.

### Enhanced dissolved oxygen concentration during fermentation

**Fig. 6:**
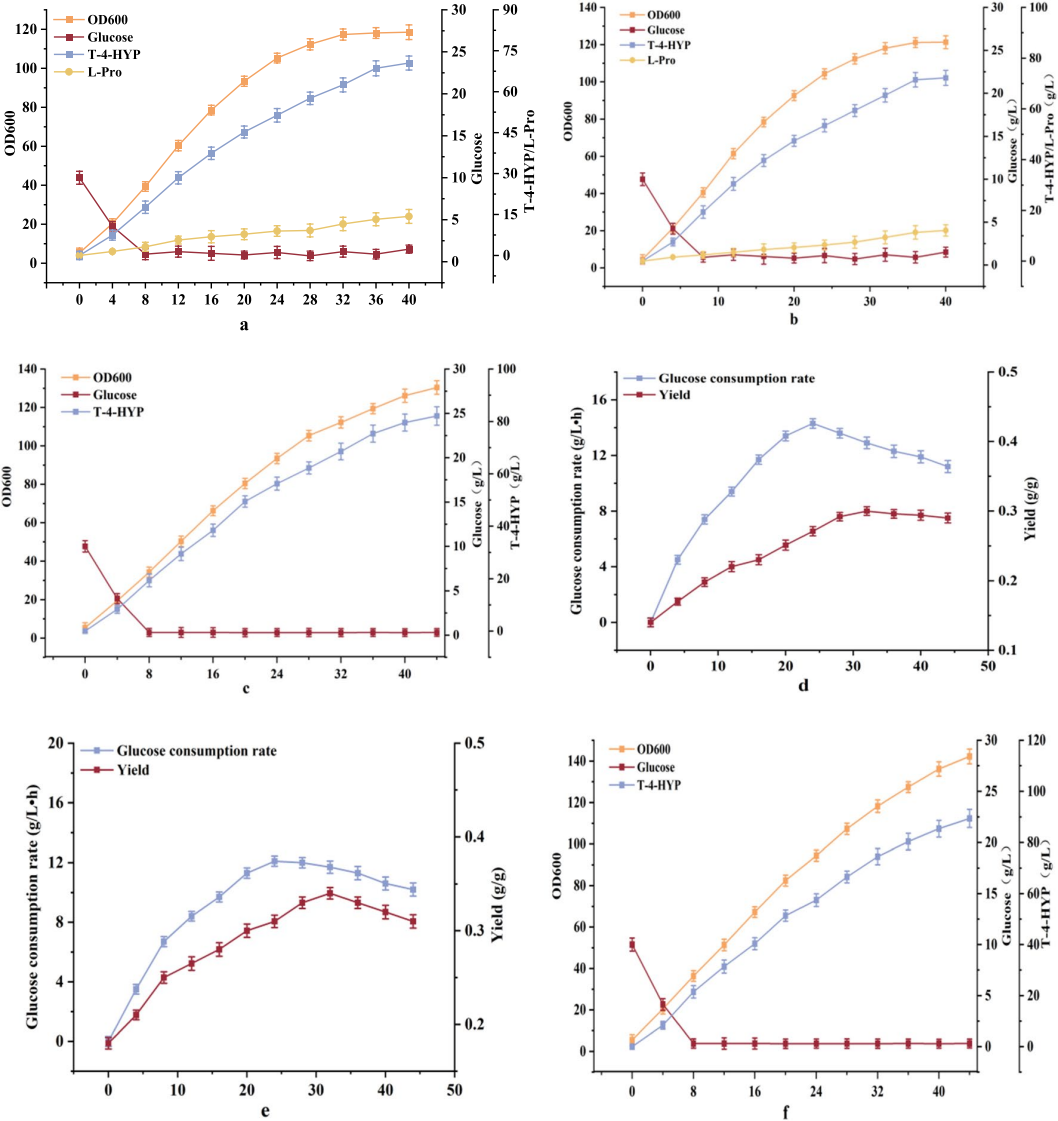
**a** The fermentation parameters of strain HYP-9; **b** fermentation parameters of strain HYP-10; **c** effect of adjusting sugar feeding rate on T-4-HYP fermentation; **d**, **e** effect of adjusting sugar feeding rate on yield; **f** effect of continuous feeding of Fe^2+^ on T-4-HYP fermentation.HYP-10, HYP-9 *yeeP*::P_*trc*_-*vgb*

Proline hydroxylase is a kind of α-ketoglutarate-dependent dioxygenase that increasing stirring can cause serious damage to the cells, which in turn leads to a decrease in the product synthesis rate [31]. Liu and Ciobanu [31, 32] added separately protease and *n*-dodecane to increase the dissolved oxygen content during the fermentation process, which greatly improved the yield of the target product. However, this not only increased the production cost, but also made the subsequent extraction and purification processes more challenging. It has been reported that Vitreoscilla hemoglobin gene (*vgb*), can efficiently transport oxygen [33]. The presence of *vgb* accelerates oxygen transfer to enhance ATP production and oxidative metabolism, and has been widely used in aerobic fermentation [34]. Therefore, in this study, *vgb* was first expressed under the control of the Ptrc promoter and integrated into the HYP-9 genome to obtain HYP-10. After 26 h of shake-flask fermentation, the yield of T-4-HYP was 16.97 g/L, which was not significantly higher than that of HYP-9. During the production of T-4-HYP in a 5 L fermenter, excessive cell growth resulted in an insufficient supply of dissolved oxygen at 6 h of fermentation. Monitoring of the fermenter data showed that the dissolved oxygen in the fermentation broth was zero at this time, and finally at 36 h of fermentation. OD and T-4-HYP production stabilized at 122.1 and 72.2, respectively (Fig. 6b). The strain HYP-9 produced 70.5 g/L of T-4-HYP (Fig. 6a). The yield of the two was not much different, and both showed L-Pro accumulations of 12.1 and 14.3 g/L, respectively. Therefore, alternative approaches must be used to solve the problem of insufficient dissolved oxygen in the fermentation broth.

During the production process in 5 L fermentor, the dissolved oxygen can be regulated to a certain extent, by sugar supplementation. The glucose addition strategy was changed from batch feeding to continuous feeding. The dissolved oxygen concentration in the fermentation broth was controlled by regulating the glucose addition rate. When the glucose concentration in the fermentation broth was controlled at 0.3 g/L, the dissolved oxygen in the fermentation broth was maintained at approximately 35% until the end of fermentation. It can be seen from Fig. 6c that the OD of the experimental group was 130.4, which was 6.7% higher than it was before optimization. The yield of T-4-HYP reached the highest value of 80.1 g/L, which was 10.9% higher than that before optimization, and L-Pro did not accumulate. Based on the comparison between Fig. 6d and e, the maximum sugar consumption rate without this feeding strategy was 14.3 g/(h·L). Using this strategy, the overall sugar consumption rate was relatively stable. At 28 h, the sugar consumption rate reached its highest levels which was 12.1 g/(h·L). The sugar consumption rate decreased during the later stages of fermentation. Although this strategy prolonged the fermentation time of the strain, the slow sugar feeding rate reduced sugar consumption by the bacteria, thereby increasing the yield of T-4-HYP. After 44 h, the final yield of 0.33 g/g was achieved.

Data are expressed as mean and error bars are expressed as standard deviation (n = 3 independent experiments). *Means 0.01 < P < 0.05, **means P < 0.01.

### The effect of Fe^2+^ on the fermentation process

Fe^2+^ is an essential trace element in cell growth and production, and plays a role in electron-transfer reactions, gene regulation, oxygen binding, and transport [5]. It is also a cofactor of the key enzyme (proline hydroxylase) in the process of T-4-HYP fermentation and participates in the hydroxylation of proline. Therefore, a practical strategy to increase the yield of T-4-HYP is to maintain Fe^2+^ in the medium at the minimum concentration required for T-4-HYP fermentation. According to Vasylkivska et al. [35], in *C. acetobutylicum*, iron is a component of the redox protein, which is an iron-sulfur protein involved in electron transfer. They participate in the formation of hydrogen by catalyzing proton reduction, and play an important role in this pathway. Alsaker et al. [36] found that Fe^2+^ plays a role in spore formation, resulting in a significant increase in the expression of the ferrous absorption system X276_13995-X276_13990-X276_13985 in the stationary phase. To ensure a sufficient supply of Fe^2+^, a key regulatory factor during the fermentation process of *E. coli* producing T-4-HYP, we chose a continuous fed-batch fermentation strategy in a 5 L bioreactor. This ensured that Fe^2+^ was maintained at a relatively stable concentration during the fermentation process, providing sufficient power for proline hydroxylase. FeSO_4_ (20 mg/L) was added to the initial medium, and FeSO_4_ was continuously fed at a rate of 3.0 mg/L/h from 10 h. The results of fermentation showed that the cells grew in the logarithmic phase from the beginning of fermentation and entered the stable phase at 28 h. The maximum yield of 89.4 g/L was obtained after 44 h of fermentation.with the highest OD600 of 75.3 at 44 h.

## Discussion

T-4-HYP has extensive applications, and its market demand continues to grow, necessitating the establishment of an efficient microbial cell factory for T-4-HYP production to meet market requirements. However, large-scale industrial production of T-4-HYP presents several challenges. These include pronounced carbon losses due to substantial metabolic diversion in *E. coli*, carbon flux competition between central metabolism and the target product synthesis pathway, and inadequate cofactor (O_2_, Fe^2+^) supplementation during the fermentation process. These factors significantly constrained the yield of T-4-HYP.

Previous efforts in metabolic engineering to enhance T-4-HYP production have predominantly focused on eliminating side pathways and screening high-efficiency prolyl 4-hydroxylases [14, 37]. While these interventions improved T-4-HYP efficacy, the carbon flux to α-ketogluterate remained far below the levels of efficient expression observed in prolyl 4-hydroxylase (P4H) production, consequently resulting in suboptimal T-4-HYP yields when compared with the theoretical values.To overcome these challenges, we reallocated carbon metabolism in the T-4-HYP biosynthetic pathway, introduced the NOG pathway and redesigned key regulatory genes. Additionally, we incorporated NADPH generation and cofactor (O_2_, Fe^2+^) supply modules, followed by systematic optimization, to construct an exemplary T-4-HYP-producing microbial strain. This strain achieved the highest yield of microbial T-4-HYP, demonstrating its promising prospects for industrial applications.

Insufficient supply of α-ketoglutaric acid has become a key bottleneck. Therefore, how to fine-tune the complex metabolic flux of the T-4-HYP biosynthetic pathway is a core challenge. Previous studies have focused on specific metabolic optimization to solve this challenge. However, compared with the previous local modifications, we systematically fine-tuned multiple metabolic flow nodes to achieve higher T-4-HYP production efficiency. Among them, we down-regulated the expression level of SucAB gene with weak promoters of different intensities, and the yield of T-4-HYP was significantly increased after down-regulation, without affecting cell growth. In addition, we introduced the xfp gene into HYP-6-1 to redirect the carbon flux of glucose to Acetyl-CoA, which improved the fermentation efficiency of T-4-HYP.The pentose-phosphate pathway (PPP) is conventionally regarded as the primary source of NADPH, a key reducing agent in cellular biosynthesis. Cai et al. [38] systematically investigated the contributions of PPP, tricarboxylic acid cycle, and hydrogenase system to NADPH production. Overexpression of zwf gene to enhance NADH levels significantly improved γ-PGA yields. Kamata et al. [39] employed gene regulation to augment NADH supply by diverting metabolic flux from the EMP pathway to the PPP through modulation of PGI gene expression. However, a limitation of this approach lies in the CO_2_ release associated with the PPP pathway, which hinders acetyl-CoA synthesis and results in a diminished product yield, rendering it unsuitable for hydroxyproline biosynthesis.we attempted to overexpress *PntAB* genes to elevate intracellular NADPH levels, and the results from shake flask experiments substantiated the effectiveness of this approach. Hence, enhancing PntAB expression during synthesis is an efficient strategy to boost T-4-HYP production.

Results from a 5 L bioreactor fermentation demonstrated that strain HYP-10 exhibits greater competitive potency in terms of efficiency, rendering industrial-scale T-4-HYP production a viable possibility.

The yield of product is an important criterion to evaluate the feasibility of microbial cell factories for industrial production. Johnsen et al. [40], found that Weimberg pathway can effectively convert xylose into α-ketoglutarate through five reactions in the next experiment, we will try to introduce this pathway to test whether the product yield can be further improved.

## Conclusion

The NOG pathway is a key acetyl-CoA supplementation pathway that significantly affects the yield of T-4-HYP. This pathway provides precursors for T-4-HYP biosynthesis, and reduces carbon loss. The highest levels of T-4-HYP production from glucose reported to date can be achieved through the following strategies: (1) construction of the T-4-HYP production base strain HYP-1; (2) strengthening the carbon flux of α-ketoglutarate in the tricarboxylic acid cycle; (3) introducing the phosphoketolase pathway (NOG pathway); (4) construction of the NADH enhancement module; and (5) continuous fed-batch fermentation strategy to strengthen the supply of cofactors (O_2_, Fe^2+^). Simultaneously, this study demonstrated that the introduction of the NOG pathway can greatly increase the yield of compounds using acetyl-CoA as a precursor. This metabolic engineering strategy can be applied to effectively produce compounds using α-ketoglutarate as a precursor.

## Materials and methods

### Chemicals and reagents

Unless otherwise stated, all chemicals and reagents were purchased from Nanjing Novozan Biotechnology Co., Ltd. Co., Ltd., China, and were of analytical grade or higher purity. Glucose monohydrate was purchased from China Xiwang Pharmaceutical Co., Ltd., and DNA polymerase was purchased from Dalian Bao Biological Co., Ltd. The oligonucleotide primers (Additional file 1: Table S1) were synthesized by Tianjin Jinweizhi Biological Co., Ltd.

### Plasmids, strains, and culture conditions

The plasmids and engineered strains used in this study are listed in Table 1. *E. coli* DH5α was used for the construction and cloning of plasmid vectors, and *E. coli* W3110 was used as the starting strain for gene manipulation. Plasmids pREDCas9 and pGRB were used in CRISPR/Cas9-mediated gene editing systems [41]. Ampicillin 100 µg/mL and spectinomycin 50 µg mL were added as required. For the expression studies, induction was achieved adding isopropyl β-d-thiogalactopyranoside (IPTG) and l-arabinose at a final concentration of 0.2 mM and 0.2% (w /v), respectively.

**Table 1:** Strains and plasmids used in this study

Strains	Characteristics	Source
*E. coli* DH5α	Host for cloning	Lab stock
*E. coli* W3110	Wild type, starting strain	Lab stock
Pro-1	*E. coli* W3110, ∆*putA*	This study
Pro-2	Pro-1, ∆*lacI*, *yghX*::P_*trc*_-*proB*_*74*_	This study
Pro-3	Pro-2, *rpH*::P_*trc*_-*proB*_*74*_	This study
Pro-4	Pro-3, *yjiT*::P_*trc*_-*proA*	This study
Pro-5	Pro-4, *yciQ*::P_*trc*_-*proC*	This study
H-1	*E. coli* W3110, P_*trc*_-*DaP4H*	This study
H-A	*E. coli* W3110, Ptrc99a	This study
HYP-A	Pro-5, Ptrc99a	This study
HYP-1	Pro-5, Ptrc99a-DaP4H	This study
HYP-2	HYP-1, ∆*ldhA*	This study
HYP-3	HYP-2, ∆*poxB*	This study
HYP-4	HYP-3, ∆*ackA*	This study
HYP-5	HYP-4, ∆*aceA*	This study
HYP-6-1	HYP-5, P_*BBa-j23109*_-*SucAB*	This study
HYP-6-2	HYP-5, P_*BBa-j231115*_-*SucAB*	This study
HYP-6-3	HYP-5, P_*BBa-j23114*_-*SucAB*	This study
HYP-7-1	HYP-6-1, *ycdN*::P_*trc*_-*Baxfp*	This study
HYP-7-2	HYP-6-1, *ycdN*::P_*trc*_-*Acxfp*	This study
HYP-7-3	HYP-6-1, ycdN::P_trc_-*Blxfp*	This study
HYP-8	HYP-7-1, P_*trc*_-*PntAB*	This study
HYP-9	HYP-8, *yjiP*::P_trc_-*PntAB*	This study
HYP-10	HYP-9, *yeeP*::P_*trc*_-*vgb*	This study
pGRB	gRNA expression vector	Lab stock
pRed-cas9	Cas9 expression vector	Lab stock
Strains	Characteristics	Source
*E. coli* DH5α	Host for cloning	Lab stock
*E. coli* W3110	Wild type, starting strain	Lab stock
pGRB	gRNA expression vector	Lab stock
pRed-cas9	Cas9 expression vector	Lab stock

### Construction of the recombinant plasmid

The recombinant plasmid was constructed by homologous recombination (Additional file 1). Taking the construction of Hyp gene overexpression plasmid Ptrc-DapP4H as an example, primers A, B, C, and D were designed using CE Design V1.03 software, and each primer had corresponding homologous sequences. The target fragment and linear vector were then amplified using primer pairs A/B and C/D, respectively. Finally, the linear vector was integrated into the target gene fragment by homologous recombination using a ClonExpress II one-step cloning kit (Vazyme Biotech, Nanjing, China), following the manufacturer’s instructions.

### Genome manipulation

The *E. coli* w3110 genome was modified using CRISPR/Cas9-mediated gene editing [42]. Here, deletion of the putA gene was used to describe the process of genome manipulation. First, a 20 bp spacer sequence was obtained using CRISPR RGEN Tools (http://www.rgenome.net/). A pair of complementary primers (PGRB-putA-S and PGRB-putA-A) was synthesized and annealed to form dsDNA containing a spacer sequence and a flanking sequence homologous to the pGRB backbone. Subsequently, dsDNA was ligated to the linearized pGRB plasmid using the ClonExpress II Seamless Cloning Kit (Vazyme, China) to construct the pGRB-putA plasmid. In addition, the upstream and downstream homologous fragments of putA were amplified using the primer pairs putA-Q1/putA-Q2 and putA-Q3/putA-Q4, and the donor DNA fragment (DNA-putA) was obtained by an overlapping polymerase chain reaction. Subsequently, DNA-putA and pGRB-putA were co-transformed into *E. coli* W3110 competent cells containing pRED-Cas9 using an Eppendorf Eporator (Eppendorf, Germany). After electroporation, the cells were cultured in 1 mL of SOC medium at 32 °C for 2 h, inoculated in LB medium containing spectinomycin and antibiotics, and cultured at 36 °C for 15 h. Positive transformants were screened by colony polymerase chain reaction and sequenced (Jinweizhi Co., Ltd.). In addition, 0.2% l-arabinose can be used to correct colony.

### Shake flask fermentation

The engineered bacteria cultured on the agar slope were transferred to a 500 mL shake flask containing a 30 mL seed medium. The shaker temperature was 36 °C, and the rotation speed was 200 rpm for 10 h. The seed medium contained (per liter) 30 g glucose, 6.0 g yeast extract, 2.0 g peptone, 0.8 g/L citric acid, 6.0 g/L yeast extract, 3.0 g/L (NH4) _2_SO_4_, 0.4 g/L MgSO_4_·7H_2_O, 5.0 mg/L MnSO_4_, 0.5 mg/L VB_(1.3.5.12)_, 1.0 mL/L Micromix, and 0.5 g/L glutamic acid. Methionine 0.5 g/L, α-ketoglutaric acid 2.0 g/L, and vitamin C 1.0 g/(8 mg) at a pH value of 7.0–7.2. The seed culture (3 mL) was used to inoculate a 500 mL shake flask containing 30 mL of fermentation medium, then cultured at 36 °C and 200 rpm for 26 h. The fermentation medium contained (per liter) glucose 30 g/L, citric acid 0.8 g/L, yeast extract 2.0 g/L, (NH4) _2_SO_4_ 3.0 g/L, MgSO_4_·7H_2_O 0.4 g/L, MnSO_4_ 5.0 mg/L, VB_(1.3.5.12)_ 0.5 mg/L, Micromix 1.0 mL/L, glutamic acid 1.5 g/L, methionine 0.5 g/L, α-ketoglutaric acid 2.0 g/L, and vitamin C 2.0 g/L. During the whole fermentation process, the a pH value of the fermentation process was maintained using ammonium hydroxide (25%, v/v) according to the color change of phenol red (8 mg, which indicated a pH value of 7.0–7.2). A sterile glucose solution (60% w/v) was supplied when the glucose in the initial culture medium was depleted to meet the demand for carbon sources for cell growth and product synthesis.

### Fed-batch fermentation in a 5 L bioreactor

An appropriate amount of agar slant culture cells was transferred to 3 L of seed medium and placed in a 5 L bioreactor (Shanghai Baoxing, China). Except for phenol red, the seed and fermentation media in the bioreactor were the same as those used in the shake flasks. When the OD600 reached 17–20, 600 mL of the seed culture was retained, and fresh fermentation medium was added immediately so that the final volume of the fermentation broth was 3 L. The pH was automatically controlled by adding ammonium hydroxide (25%, v/v) and temperature was maintained at 36 °C during the entire fermentation process. The dissolved oxygen was maintained above 25% by changing the stirring speed and aeration rate. When the initial glucose was depleted, an appropriate amount of sterile glucose solution (80%, w/v) was added, and the glucose concentration was maintained below 5 g/L.

### Analytical methods

The absorbance was measured at 600 nm using an ultraviolet/visible spectrophotometer (UV1800, Shanghai Jinghua Technology Instrument Co., Ltd., Shanghai, China) to detect cell growth status. Glucose concentration was determined using a biosensor analyzer (SBA-40E, Jinan Yanhe Biotechnology Co., Ltd., Shandong, China). The standards and samples of T-4-HYP and L-Pro were quantified using high-performance liquid chromatography (Shimadzu). An Agilent reverse TC-C18 (2) chromatographic column was used, and the column temperature was 25 °C. The mobile phase was an acetonitrile/2 sodium acetate buffer (pH 6.5) (1:1, v/v) at a flow rate of 1 mL/min. The NAD(P)H and NAD(P)+ levels of the cells were detected using NADPH/NADP+ and NADH/NAD+ analysis kits with WST-8 (S0179 and S0175; Beyotime, China). Acetate and α-ketoglutaric acid was detected by HPLC (Shimadzu, Japan) using an Aminex hx-87 h chromatographic column (Bio-Rad, CA, USA) and a refractive index detector (RID-20 A, Shimadzu, Japan). The column temperature was maintained at 37 °C, 5 mM sulfuric acid was used as the mobile phase, and the flow rate was maintained at 0.5 mL/min.

### Statistical analysis

Data are expressed as mean and error bars are expressed as standard deviation (n = 3 independent experiments). *Means 0.01 < P < 0.05, **means P < 0.01.

### Supplementary Information

**Additional file 1: Table S1.** Primers used for strain construction in this study.

## Data Availability

All data generated or analyzed during this study are included in this article and its Additional file.
