# Supplementary material for: Optimization of trans-4-hydroxyproline synthesis pathway by rearrangement center carbon metabolism in Escherichia coli
Source: Microb Cell Fact. 2023 Nov 20;22:240. doi: 10.1186/s12934-023-02236-6 (PMC10659092; doi:10.1186/s12934-023-02236-6)
Supplement: Supplementary file 1 — Additional file 1: Table S1. Primers used for strain construction in this study. [file 12934_2023_2236_MOESM1_ESM.docx]

**Additional file 1**

# Optimization of trans-4-hydroxyproline synthesis pathway by rearrangement center carbon metabolism in Escherichia coli

Yu Gong^1,2^, Ruiqi Wang^1,2^, Ling Ma^1,2^, Shuo Wang ^1,2^, Changgeng Li ^1,2^，Qingyang Xu^1,2*^

1 *College of Biotechnology, Tianjin University of Science & Technology, Tianjin, 300457, P. R. China.*

2 Key *Laboratory of Industrial Fermentation Microbiology, Ministry of Education, Tianjin University of Science & Technology, Tianjin, 300457,* *P. R. China.*

**Table S1** Primers used for strain construction in this study.

Table S1. Primers used for strain construction

| Primers | Sequence (5’→ 3’) |
| --- | --- |
| putA-Q-1 | GCGTATTCCCGACAAAGCC |
| putA-Q-2 | TAATGGACGGTGGGTTTCGTCATAGATGCCACGACCGTTAGA |
| putA-Q-3 | TCTAACGGTCGTGGCATCTATGACGAAACCCACCGTCCATTA |
| putA-Q-4 | AACAACGCTGACCCGCAC |
| pGRB-putA-S | AGTCCTAGGTATAATACTAGTGTGCGTCGCCTGCTGGAAAAGTTTTAGAGCTAGAA |
| pGRB-putA-A | TTCTAGCTCTAAAACTTTTCCAGCAGGCGACGCACACTAGTATTATACCTAGGACT |
| lacI-Q-1 | GACTATCAACTGGCACGGGAA |
| lacI-Q-2 | CGATTTGCTGGTGACCCAAGCGGGAAACGGTCTGATAAG |
| lacI-Q-3 | CTTATCAGACCGTTTCCCGCTTGGGTCACCAGCAAATCG |
| lacI-Q-4 | TTTTCACCAGTGAGACGGGC |
| pGRB-lacI-S | AGTCCTAGGTATAATACTAGTGCCACGTTTCTGCGAAAACGGTTTTAGAGCTAGAA |
| pGRB-lacI-A | TTCTAGCTCTAAAACCGTTTTCGCAGAAACGTGGCACTAGTATTATACCTAGGACT |
| yghX-UP-S | GCGCAACGTAGAACAGGAATT |
| yghX-UP-A | TGTGTGAAATTGTTATCCGCTCACAATTCCACACATTATACGAGCCGGATGATTAATTGTCAAGATTGAAGCGCCTTTACTACTCC |
| yghX-DN-S | AAAGACTGGGCCTTTCGTTTTATCTGTTGTTTGTCGGTGAACGCTCTCCTGAGTAGGACAAATGTCATAGTAATCCAGCAACTCTTGTG |
| yghX-DN-A | GAGCAGGTATTTACGTGAACCG |
| PGRB-yghX-S | AGTCCTAGGTATAATACTAGTGGTGCCTGACGACCATAAAAGTTTTAGAGCTAGAA |
| PGRB-yghX-A | TTCTAGCTCTAAAACTTTTATGGTCGTCAGGCACCACTAGTATTATACCTAGGACT |
| rpH-UP-S | ATAGCGCAGGGTACATTCCACT |
| rpH-UP-A | AATTGTTATCCGCTCACAATTCCACACATTATACGAGCCGGATGATTAATTGTCAACCTTCTTCAATAGAGGCGGTACA |
| rpH-DN-S | AAAGACTGGGCCTTTCGTTTTATCTGTTGTTTGTCGGTGAACGCTCTCCTGAGTAGGACAAATTGCCGCAGAGACCGACAT |
| rpH-DN-A | ACAGCGGTTGTGGTGGCA |
| pGRB-rpH-S | AGTCCTAGGTATAATACTAGTGGCTGGATCACCGCAGAGTAGTTTTAGAGCTAGAA |
| pGRB-rpH-A | TTCTAGCTCTAAAACTACTCTGCGGTGATCCAGCCACTAGTATTATACCTAGGACT |
| putA-Q-1 | GCGTATTCCCGACAAAGCC |
| putA-Q-2 | TAATGGACGGTGGGTTTCGTCATAGATGCCACGACCGTTAGA |
| putA-Q-3 | TCTAACGGTCGTGGCATCTATGACGAAACCCACCGTCCATTA |
| putA-Q-4 | AACAACGCTGACCCGCAC |
| pGRB-putA-S | AGTCCTAGGTATAATACTAGTGTGCGTCGCCTGCTGGAAAAGTTTTAGAGCTAGAA |
| pGRB-putA-A | TTCTAGCTCTAAAACTTTTCCAGCAGGCGACGCACACTAGTATTATACCTAGGACT |
| lacI-Q-1 | GACTATCAACTGGCACGGGAA |
| lacI-Q-2 | CGATTTGCTGGTGACCCAAGCGGGAAACGGTCTGATAAG |
| lacI-Q-3 | CTTATCAGACCGTTTCCCGCTTGGGTCACCAGCAAATCG |
| lacI-Q-4 | TTTTCACCAGTGAGACGGGC |
| pGRB-lacI-S | AGTCCTAGGTATAATACTAGTGCCACGTTTCTGCGAAAACGGTTTTAGAGCTAGAA |
| pGRB-lacI-A | TTCTAGCTCTAAAACCGTTTTCGCAGAAACGTGGCACTAGTATTATACCTAGGACT |
| yghX-UP-S | GCGCAACGTAGAACAGGAATT |
| yghX-UP-A | TGTGTGAAATTGTTATCCGCTCACAATTCCACACATTATACGAGCCGGATGATTAATTGTCAAGATTGAAGCGCCTTTACTACTCC |
| yghX-DN-S | AAAGACTGGGCCTTTCGTTTTATCTGTTGTTTGTCGGTGAACGCTCTCCTGAGTAGGACAAATGTCATAGTAATCCAGCAACTCTTGTG |
| yghX-DN-A | GAGCAGGTATTTACGTGAACCG |
| PGRB-yghX-S | AGTCCTAGGTATAATACTAGTGGTGCCTGACGACCATAAAAGTTTTAGAGCTAGAA |
| PGRB-yghX-A | TTCTAGCTCTAAAACTTTTATGGTCGTCAGGCACCACTAGTATTATACCTAGGACT |
| rpH-UP-S | ATAGCGCAGGGTACATTCCACT |
| rpH-UP-A | AATTGTTATCCGCTCACAATTCCACACATTATACGAGCCGGATGATTAATTGTCAACCTTCTTCAATAGAGGCGGTACA |
| rpH-DN-S | AAAGACTGGGCCTTTCGTTTTATCTGTTGTTTGTCGGTGAACGCTCTCCTGAGTAGGACAAATTGCCGCAGAGACCGACAT |
| rpH-DN-A | ACAGCGGTTGTGGTGGCA |
| pGRB-rpH-S | AGTCCTAGGTATAATACTAGTGGCTGGATCACCGCAGAGTAGTTTTAGAGCTAGAA |
| pGRB-rpH-A | TTCTAGCTCTAAAACTACTCTGCGGTGATCCAGCCACTAGTATTATACCTAGGACT |
| putA-Q-1 | GCGTATTCCCGACAAAGCC |
| putA-Q-2 | TAATGGACGGTGGGTTTCGTCATAGATGCCACGACCGTTAGA |
| putA-Q-3 | TCTAACGGTCGTGGCATCTATGACGAAACCCACCGTCCATTA |
| putA-Q-4 | AACAACGCTGACCCGCAC |
| pGRB-putA-S | AGTCCTAGGTATAATACTAGTGTGCGTCGCCTGCTGGAAAAGTTTTAGAGCTAGAA |
| pGRB-putA-A | TTCTAGCTCTAAAACTTTTCCAGCAGGCGACGCACACTAGTATTATACCTAGGACT |
| lacI-Q-1 | GACTATCAACTGGCACGGGAA |
| lacI-Q-2 | CGATTTGCTGGTGACCCAAGCGGGAAACGGTCTGATAAG |
| lacI-Q-3 | CTTATCAGACCGTTTCCCGCTTGGGTCACCAGCAAATCG |
| lacI-Q-4 | TTTTCACCAGTGAGACGGGC |
| pGRB-lacI-S | AGTCCTAGGTATAATACTAGTGCCACGTTTCTGCGAAAACGGTTTTAGAGCTAGAA |
| pGRB-lacI-A | TTCTAGCTCTAAAACCGTTTTCGCAGAAACGTGGCACTAGTATTATACCTAGGACT |
| yghX-UP-S | GCGCAACGTAGAACAGGAATT |
| yghX-UP-A | TGTGTGAAATTGTTATCCGCTCACAATTCCACACATTATACGAGCCGGATGATTAATTGTCAAGATTGAAGCGCCTTTACTACTCC |
| yghX-DN-S | AAAGACTGGGCCTTTCGTTTTATCTGTTGTTTGTCGGTGAACGCTCTCCTGAGTAGGACAAATGTCATAGTAATCCAGCAACTCTTGTG |
| yghX-DN-A | GAGCAGGTATTTACGTGAACCG |
| PGRB-yghX-S | AGTCCTAGGTATAATACTAGTGGTGCCTGACGACCATAAAAGTTTTAGAGCTAGAA |
| PGRB-yghX-A | TTCTAGCTCTAAAACTTTTATGGTCGTCAGGCACCACTAGTATTATACCTAGGACT |
| rpH-UP-S | ATAGCGCAGGGTACATTCCACT |
| rpH-UP-A | AATTGTTATCCGCTCACAATTCCACACATTATACGAGCCGGATGATTAATTGTCAACCTTCTTCAATAGAGGCGGTACA |
| rpH-DN-S | AAAGACTGGGCCTTTCGTTTTATCTGTTGTTTGTCGGTGAACGCTCTCCTGAGTAGGACAAATTGCCGCAGAGACCGACAT |
| rpH-DN-A | ACAGCGGTTGTGGTGGCA |
| pGRB-rpH-S | AGTCCTAGGTATAATACTAGTGGCTGGATCACCGCAGAGTAGTTTTAGAGCTAGAA |
| pGRB-rpH-A | TTCTAGCTCTAAAACTACTCTGCGGTGATCCAGCCACTAGTATTATACCTAGGACT |
| proB-trc-UP | CATCCGGCTCGTATAATGTGTGGAATTGTGAGCGGATAACAATTTCACACAGGAAACAGACCATGCGTGAGCGCATCTCC |
| proB-trc-DN | TTCACCGACAAACAACAGATAAAACGAAAGGCCCAGTCTTTCGACTGAGCCTTTCGTTTTATTTGTTACGCGCGGCTGGC |
| yjiT-UP-S | AATAGTTGTTGCCGCCTGAGT |
| yjiT-UP-A | TGTGTGAAATTGTTATCCGCTCACAATTCCACACATTATACGAGCCGGATGATTAATTGTCAAAAAACAGGCAGCAAAGTCCC |
| yjiT-DN-S | AAAGACTGGGCCTTTCGTTTTATCTGTTGTTTGTCGGTGAACGCTCTCCTGAGTAGGACAAATAAGCACTACCTGTGAAGGGATGT |
| yjiT-DN-A | CAGGGCTTCCACAGTCACAAT |
| PGRB-yjiT-S | AGTCCTAGGTATAATACTAGTAGGGATTATGAACGGCAATG  GTTTTAGAGCTAGAA |
| PGRB-yjiT-A | TTCTAGCTCTAAAACCATTGCCGTTCATAATCCCTACTAGTATTATACCTAGGACT |
| proA-trc-UP | TCCGGCTCGTATAATGTGTGGAATTGTGAGCGGATAACAATTTCACACAGGAAACAGACCATGCTGGAACAAATGGGCAT |
| proA-trc-DN | CAAACAACAGATAAAACGAAAGGCCCAGTCTTTCGACTGAGCCTTTCGTTTTATTTGTTACGCACGAATGGTGTAATCAC |
| yciQ-UP-S | TTACTTGAAGCATTGGGCGAAC |
| yciQ-UP-A | AATTGTTATCCGCTCACAATTCCACACATTATACGAGCCGGATGATTAATTGTCAACCAGTCAAGATGCCAGGGTTC |
| yciQ-DN-S | AAAGACTGGGCCTTTCGTTTTATCTGTTGTTTGTCGGTGAACGCTCTCCTGAGTAGGACAAATGTCTGACAAGAACCAGCAAATCCT |
| yciQ-DN-A | ATAGCTTCACCGTGGGCATAAC |
| PGRB-yciQ-S | AGTCCTAGGTATAATACTAGTAAACAACGTTTCTTGCCTCAGTTTTAGAGCTAGAA |
| PGRB-yciQ-A | TTCTAGCTCTAAAACTGAGGCAAGAAACGTTGTTTACTAGTATTATACCTAGGACT |
| proC-trc-UP | CGGATAACAATTTCACACAGGAAACAGACCGTGGGAACCATGACAACAATTGCT |
| proC-trc-DN | TCTTTCGACTGAGCCTTTCGTTTTATTTGCTAGCGCTTTCCGAGTTCTTCA |
| H-1-UP | AGGAAACAGACCATGGAATTCATGCTGACCCCGACCGAA |
| H-1-DN | TGCCTGCAGGTCGACTCTAGATTAAACCGGCTGAGCCAGAG |
| ldhA-Q-1 | AACGAGTCCTTTGGCTTTG |
| ldhA-Q-2 | ACTTGGATACGGATCGAACAGAAGTTAGCATCACGGGTAC |
| ldhA-Q-3 | GTACCCGTGATGCTAACTTCTGTTCGATCCGTATCCAAGT |
| ldhA-Q-4 | CGCCTCCAGATTGCTTA |
| pGRB-ldhA-S | AGTCCTAGGTATAATACTAGTATGTATGGCAAAACGGCAAA GTTTTAGAGCTAGAA |
| pGRB-ldhA-A | TTCTAGCTCTAAAACTTTGCCGTTTTGCCATACATACTAGTATTATACCTAGGACT |
| poxB-Q-1 | GAAGCACAACTTAGCGGAGAA |
| poxB-Q-2 | CGCTTGCCGTTCATTTTCCCGGCAAACTCAACTAACT |
| poxB-Q-3 | AGTTAGTTGAGTTTGCCGGGAAAATGAACGGCAAGCG |
| poxB-Q-4 | CGGTGGAATGGCTAACTCTT |
| pGRB-poxB-S | AGTCCTAGGTATAATACTAGTTACGATAATCCGTATGATGT GTTTTAGAGCTAGAA |
| pGRB-poxB-A | TTCTAGCTCTAAAACACATCATACGGATTATCGTAACTAGTATTATACCTAGGACT |
| ackA-Q-1 | GTGATATCGATCCGGCGATCATCACTGCGGTAGTTCTTCACTGAAATTT |
| ackA-Q-2 | GTTGTAAGGCAGGGCGTAGAG |
| ackA-Q-3 | AAATTTCAGTGAAGAACTACCGCAGTGATGATCGCCGGATCGATATCAC |
| ackA-Q-4 | TTGCGCGATAACCAGTTCTTC |
| pGRB-ackA-S | AGTCCTAGGTATAATACTAGTACCTGCCACCTGGGCAACGGGTTTTAGAGCTAGAA |
| pGRB-ackA-A | TTCTAGCTCTAAAACCCGTTGCCCAGGTGGCAGGTACTAGTATTATACCTAGGACT |
| aceA-Q-1 | ACCAAAGCCTTGTTCCG |
| aceA-Q-2 | TGCTGTGGATACCTGCCACCTACAAATTGAGTTATGTTCATGCCATCCCGACAGATAGAC |
| aceA-Q-3 | GTCTATCTGTCGGGATGGCATGAACATAACTCAATTTGTAGGTGGCAGGTATCCACAGCA |
| aceA-Q-4 | GCATTTAGCGCCCTCATC |
| pGRB-aceA-S | AGTCCTAGGTATAATACTAGTGTCGTGCCGATCAGATCCAAGTTTTAGAGCTAGAA |
| pGRB-aceA-S | TTCTAGCTCTAAAACTTGGATCTGATCGGCACGACACTAGTATTATACCTAGGACT |
| SucAB-UP-S | ATCATGAACTGCGTCAGTGTATGTC |
| SucAB-UP-A（BBa_j23109） | GGTCTGTTTCCTGCTAGCACAGTCCCTAGGACTGAGCTAGCTGTAAACGTAGTGGCTCGCGAAGGA |
| SucAB-DN-S（BBa_j23109） | TTTACAGCTAGCTCAGTCCTAGGGACTGTGCTAGCAGGAAACAGACCATGCAGAACAGCGCTTTGAAA |
| SucAB-UP-A（BBa_j23115） | GCTAGCATTGTACCAAGGGCTGAGCTAGCTATAAACGTAGTGGCTCGCGAAGGA |
| SucAB-DN-S（BBa_j23115） | TTTATAGCTAGCTCAGCCCTTGGTACAATGCTAGCATGCAGAACAGCGCTTTGAAA |
| SucAB-UP-A（BBa_j23114） | GCTAGCATTGTACCTAGGACTGAGCTAGCCATAAACGTAGTGGCTCGCGAAGGA |
| SucAB-DN-S（BBa_j23114） | TTTATGGCTAGCTCAGTCCTAGGTACAATGCTAGCATGCAGAACAGCGCTTTGAAA |
| SucAB-DN-A | ATCGGCCACTTTATCTTGCTGC |
| ycdN-UP-S | GATTTTGACGCCACCAACACC |
| ycdN-UP-A | GTTATCCGCTCACAATTCCACACATTATACGAGCCGGATGATTAATTGTCAACCAATCCACATCACACAATCCATC |
| ycdN-DN-S | CTGGGCCTTTCGTTTTATCTGTTGTTTGTCGGTGAACGCTCTCCTGAGTAGGACAAATGAAGGGATTTTTGGCTATCAGG |
| ycdN-DN-A | CATATCGTATTCGCCAGGCTG |
| xfP-trc-UP | TCGTATAATGTGTGGAATTGTGAGCGGATAACAATTTCACACAGGAAACAGACCGAATTCATGACCTCTCCAGTCATCG |
| xfP-trc-DN | ACAAACAACAGATAAAACGAAAGGCCCAGTCTTTCGACTGAGCCTTTCGTTTTATTTGGGATCCTTATTCATTGTCGCCA |
| PutAB1-UP-S | ATGCGAATTGGCATACCAAGA |
| PutAB1-UP-A | CGGCTCGTATAATGTGTGGAATTGTGAGCGGATAACAATTTCACACAGGAAACAGACCAATTAGTAACAAACGCCCTATAACGT |
| PutAB1-DN-S | GACAAACAACAGATAAAACGAAAGGCCCAGTCTTTCGACTGAGCCTTTCGTTTTATTTGATGCGAATTGGCATACCAAGA |
| PutAB1-DN-A | TTTTTGCATTAATTCCGGATTCT |
| PGRB-PntAB1-S | AGTCCTAGGTATAATACTAGTGCTTGTGTGGCTCCTGACACGTTTTAGAGCTAGAA |
| PGRB-PntAB1-A | TTCTAGCTCTAAAACGTGTCAGGAGCCACACAAGCACTAGTATTATACCTAGGACT |
| Yjip-UP1-S | GCCATACCGCCAGCAAGAT |
| Yjip-UP1-A | GCAAGGCGAAGGATTATTTTTGCAGATATTCCCCTTTCCACC |
| Z-1-S | GGTGGAAAGGGGAATATCTGCAAAAATAATCCTTCGCCTTGC |
| Z-1-A | GCCCCAAGGGGTTATGCTAGCCTACAAATTGAGTTATGTTCATTTAAATATGATGTTGTTCAGTTAATTTTTGCGGAACATTTTCA |
| Yjip-DN1-S | CTGAACAACATCATATTTAAATGAACATAACTCAATTTGTAGGCTAGCATAACCCCTTGGGGCGACGGATGACAAACGCAAAGC |
| Yjip-DN1-A | AAAGGCGGATTTTTACTGTGGA |
| Yjip-up2-S | CGTTGCGCCGAAAGAATT |
| Yjip-up2-A | GCTTTGCGTTTGTCATCCGTCTTACAGAGCTTTCAGGATTGCAT |
| Yjip-DN2-S | ATGCAATCCTGAAAGCTCTGTAAGACGGATGACAAACGCAAAGC |
| Yjip-DN2-A | AAAGGCGGATTTTTACTGTGGA |
| yeeP-UP-S | GGTCAGGAGGTAACTTATCAGCG |
| yeeP-UP-A | CCACACATTATACGAGCCGGATGATTAATTGTCAAATGGCAGGGCTCCGTTTT |
| yeeP-DN-S | TTCGTTTTATCTGTTGTTTGtcggtgaacgctctcctgagtaggacaaatGAACTGGATTTTCTTCTGAACCTGT |
